# Isokinetic and Electromyographic Properties of Muscular Endurance in Short and Long-Term Type 2 Diabetes

**DOI:** 10.5539/gjhs.v8n8p210

**Published:** 2015-12-17

**Authors:** Boshra Hatef, Ali Ghanjal, Gholam Hossein Meftahi, Ahmadreza Askary-Ashtiani

**Affiliations:** 1 Neuroscience Research Center, Baqiyatallah University of Medical Sciences, Tehran, Iran; 2 Health Management Research Centre, Department of Physical Medicine and Rehabilitation, Baqiyatallah University of Medical Sciences, Tehran, Iran; 3 Health Promotion Research Center, Zahedan University of Medical Sciences, Zahedan, Iran

**Keywords:** type 2 diabetes mellitus, muscular endurance, isokinetic, knee muscles

## Abstract

**Background:**

Patients with type 2 diabetes mellitus (T2DM) are subject to progressive reduction of muscle mass and strength. The aim of this study was to assess muscle forces and electromyography (EMG) indices in short and long-term diabetes during an isokinetic exercise.

**Methods:**

The peak torque, work, mean power frequency (MPF) and root mean square (RMS) of knee flexors and extensors during 40 isokinetic knee extension-flexion repetitions with a velocity of 150 degree/s were recorded. 18 patients with less than 10 years with T2DM and 12 patients with equal and more than 10 years of disease were compared with 20 gender, body mass index, physical activity and peripheral circulation matched healthy controls.

**Results:**

The fatigue index and slope of line across the peak torque values of the knee flexor indicate that patients with long-term T2DM were significantly more resistant to fatigue in comparison with the two other groups (p<0.009). Whereas the MPF decrease during isokinetic protocol interact with grouping in the medial hamstring (p<0.042), but it was independent to groups in other muscles (p<0.0001). The increase of RMS after fatigue protocol interacted with sex for the medial hamstring and vastus lateralis (p<0.039) and interacted with group for the extensor muscles (p<0.045).

**Discussion & Conclusion:**

It seems that long-term T2DM cause some neuromuscular adaptations to maintain knee flexor muscle performance during functional activity especially postural control.

## 1. Introduction

Muscle cell resistance to insulin, lipid metabolic inflexibility, and neuromuscular insufficiency is the primary causes of poor muscle strength and quality. The muscle weakness was more seen in lower limb than upper limb in the T2DM. Strength reduction has been associated with increased neuropathy, glycated hemoglobin A1c (HbA1c) and duration of diabetes (Andersen, 1998; Andersen, Nielsen, Mogensen, & Jakobsen, 2004; Andersen, Poulsen, Mogensen, & Jakobsen, 1996; Bokan, 2011; Halvatsiotis, Short, Bigelow, & Nair, 2002; IJzerman, 2012; Park et al., 2006; Roden, 2005; Sayer et al., 2005). However, there are not enough evidences to support diabetes-related fatigue (Andersen, 1998; Fritschi & Quinn, 2010; Halvatsiotis et al., 2002; IJzerman, 2012). In the study of Andersen et al, 44 type 2 diabetes mellitus (T2DM) patients with more than 20 years of disease were compared with 44 matched-healthy subjects. The results showed that the healthy subjects were fatigued more than patients during 30 maximal isokinetic repetitions of knee and ankle. The endurance index was not correlated to the severity of neuropathy and to the metabolic control (blood glucose and HbA1c) (Andersen, 1998). In contrast, two other isokinetic studies revealed that fatigue index in patients with T2DM was lower in the knee flexor and extensor in comparison with the healthy subjects (Halvatsiotis et al., 2002; IJzerman, 2012). It is established that the percentage of type of muscle fibers has an important role in the fatigued muscle (Zierath & Hawley, 2004). Not only sarcopenia and loss of muscle quality were observed in T2DM patients (Park et al., 2007; Zierath & Hawley, 2004), but also a decrease in proportion of fatigue-resistant type 1 muscle fibers and an increase in proportion of the fatigue-resistant type 2b muscle fibers were seen (MÅrin, Andersson, Krotkiewski, & Björntorp, 1994; Murakami et al., 2012; Oberbach et al., 2006). It is expected that the endurance capacity changes over time in parallel to morphological and functional alternations (Zierath & Hawley, 2004). Exercise has an important role in the control and treatment of diabetes. The researcher has tried to find more effective exercise protocol to control of diabetes. investigation of muscle capacity can be helpful to design exercise better (Colberg et al., 2010). However, no study has yet investigated the effects of diabetes with different duration on the endurance properties. Also there is no evidence to assess simultaneous measurement of the kinesiological electromyography (EMG) parameters and muscle force in T2DM patients. Taking into account the above-mentioned considerations, the aim of this study was to investigate the endurance indices based on isokinetic and EMG parameters of knee flexors and extensors during intensive short-term exercise in the patients with less and equal/more than 10 years of T2DM compared to healthy subjects that were matched with patients for the purpose of controlling the confounding factor such as age, gender, body mass index (BMI), physical activity index (PAI) and ankle brachial index (ABI) (Harbo, Brincks, & Andersen, 2012; IJzerman, 2012; Lanza & Nair, 2009; Lindle et al., 1997; Sreekumar R).

## 2. Materials and Methods

### 2.1 Subjects

Voluntary, thirty T2DM patients were recruited from internal medicine and endocrine centers. The included patients were: adults between 25-70 years old, without severe or uncontrolled cardiac disease diagnosed by a specialist, no intermittent foot claudication and ulceration, no muscular disorders or rheumatoid arthritis and 1.5>ABI> 0.9. Patients were categorized into two groups according to the duration of diagnosed diabetes by specialist. Eighteen patients with less than 10 years of disease were placed in the short-term T2DM group and twelve patients with equal or more than 10 years of disease were placed in the long-term T2DM group. The healthy control group consisted of twenty subjects who matched in terms of gender, BMI, ABI and PAI with the two diabetic groups. Inclusion criteria and an HbA1c<6 were took into account for healthy control group. Written informed consent was obtained from all participants. The protocol was approved by the medical ethics committee of the Tarbiat Modares University.

### 2.2 Procedure

HbA1c and FBS by blood sample and ABI by Doppler ultrasound in the supine position from the dominant leg were measured in all participants. The ABI is calculated by dividing the systolic blood pressure at the ankle by the systolic blood pressure at the ipsilateral arm. Those who were in normal range between 0.91-1.3 were entered into the study (Ena, Lozano, Verdu, Argente, & Gonzalez, 2011). ABI in the normal range indicated that no peripheral circulation disorder existed. Because we measured the muscle performance, the severity of physical activity should be evaluated. The level of physical activity was measured by scoring of PAI. For each person, the total score was obtained by multiplying intensity, duration and frequency of physical activity. Then the score was matched with physical activity category (Smith, Gallagher, Hays, Goss, & Robertson, 2012). The results showed that all participants had been categorized in the sedentary, poor and fair categories. The patients were asked to take the prescribed diabetic medications routinely and were recruited to the test session in the afternoon. The brachial pressure and finger blood glucose were measured before the test.

### 2.3 Isokinetic Protocol

Isokinetic dynamometer measured muscle performance. Repetitive concentric of knee extension/flexion were performed with an isokinetic dynamometer (HUMAC NORM, USA) for the dominant leg which was determined by the leg preferred for kicking a ball. All subjects received instructions about the procedure and performed a warm-up session which is consisted of twice quadriceps and hamstring stretching for 30 seconds and 5 minutes of free load ergonomic cycle. Participants were sited in an 85 degree hip flexion and 0-75 degree knee flexion (Larsson, Karlsson, Eriksson, & Gerdle, 2003). To stabilize the pelvis and thighs, two straps were used. Then the center of rotation of the knee joint was aligned with the rotation center of the dynamometer. The lower leg was strapped to the dynamometer lever arm by using a calf pad, 5 centimeters proximal to the lateral malleolus. The settings of the chair and lever arm for each subject were adjusted by calibrations of the instrument. Five trials of submaximal isokinetic of knee extension and flexion were done. The experiment was started after 2 minutes of resting. The subjects were instructed to push and pull “as hard and fast as possible” through the full available range of motion at every repetition without holding their breath and resting. Forty repetitions of isokinetic knee flexion-extension cycles at 150 degree/s were used. The heart rate was controlled with a finger heart beat controller device attached to the middle finger of the opposite hand during the test.

### 2.4 EMG Recording

Concurrently with isokinetic recording, surface EMG signals were recorded from the vastus lateralis, vastus medialis, and long head of biceps femoris and medal hamstring muscles of dominant leg. The EMG signals reflected the changes of motor unit behavior. Two features of EMG are amplitude and frequency that were calculated in this study. The electrodes were positioned in accordance to SENIAM (Non-Invasive Assessment of Muscles) recommendations (Hermens, Freriks, Disselhorst-Klug, & Rau, 2000). The subjects’ skin was shaved and cleaned with an 70% alcohol solution and two recording circle Ag/AgCl sensors (Telectrode, Bio Protech Inc., Korea, Diameter of 23 mm) for each muscle placed 20 mm apart (center to center distance) on the skin. A reference electrode was applied to the ipsilateral unlar styloid process. A bipolar multi-channel EMG amplifier (Bio-Signal Pack, Bayamed Co. www.bayamed.com) (Common Mode Rejection Ratio: 120 dB, Input Impedance: 10M Ω, bandwidth 200 KHz, gain: 1000) was used to register the surface EMG activity. All signals were sampled at 2460 Hz. The digital signals were converted by means analogue-to-digital converter and stored. LabVIEW Advanced Signal Processing Toolkit 10.0.0 was used for signal processing.

Then the sampled EMG signals were digitally band-pass filtered from 10 to 500 Hz. The frequency content was computed for each window using Fast Fourier Transform method. The tests were conducted for 250 msec long analysis frames with %50 overlap, using Hamming window. The mean power frequency (MPF) as a frequency feature of EMG was calculated. The root mean square (RMS) (µV) as an amplitude feature was calculated using a moving window with the same method. Each window is defined as recruited muscle activity between onset and off-set points determined based on baseline activity plus/minus 2 times the standard deviation (SD) and confirmed by visual detection.

### 2.5 Data Analysis

The amount of peak torque (Newton meter) and peak work (Newton meter× Distance) per repetition was calculated by isokinetic instrument’s software. The maximal concentric peak torque (CMPT) of flexion and extension was maximal recorded peak torque from 40 repetitions. It was divided to weight for normalization (IJzerman, 2012; Sayer et al., 2005). The peak torque of 40 repetitions was normalized to maximal peak torque and the slope of line across these 40 points was measured and introduced to slope value (Halvatsiotis et al., 2002). The fatigue index was calculated as the ratio of the mean work of the last 5 repetitions to the mean work of the highest, five consecutive repetitions within the first fifteen repetitions (IJzerman, 2012). RMS and MPF were calculated for the three terminal and initial tandem repetitions. The average of initial and terminal EMG parameters was defined. The activity of vastus lateralis, vastus medialis muscles were met with extension torques and long head of biceps femoris and medal hamstring muscles were met with flexion torques.

### 2.6 Statistical Analysis

The differences of distribution of gender and PAI between groups were assessed by Chi-squared analysis. The ANOVA procedure was used to determine the baseline differences of age, length, weight, BMI, HbA1c, ABI, FBS and blood glucose test between groups. Test of Normality and homogeneity of variances were performed for all variables. Two-way ANOVA and fisher’s least significant difference (LSD) test were carried out to analysis the difference of fatigue index and slope whereas as group and gender were main factors. Two-way ANCONA was used to compare the CMPT in the three groups and two genders while the age was considered as covariate. Three-way mixed model ANOVA and LSD Post Hoc test were performed to analyze the difference of EMG variables in different genders and groups. Pearson correlation analysis was used to detect the correlation of isokinetic and EMG variables with blood factors such as HbA1c, FBS and finger glucose test (blood sugar). P values less than 0.05 were considered as “statistically significant”. The SPSS software version 21 was used for analysis.

## 3. Results

We included 18 subjects with diabetic duration less than 10 years, 12 subjects with diabetic duration equal and more than 10 years and 20 healthy control subjects. Demographic and blood parameters are presented in Table 1, diabetic subjects with good to moderate blood glucose control were included in this study. The three groups were matched to each other in regards to gender, BMI, PAI and ABI. The healthy control group was 10 years younger than long-term T2DM group (P=0.02). HbA1c, FBS and glucose tests of the healthy control group were lower than the two diabetic groups (P<0.0001), while the two diabetic groups were not different significantly.

**Table 1:** Demographic and blood characters of three groups and P value

	Healthy control (N=19)	Short-term T2DM (N=18)	Long-term T2DM (N=12)	P value
Number of cases (Female/Male)	20 (10/10)	18 (9/9)	12 (6/6)	1
Age (years)	49.55±10	52.11±9.2	59.17±7.1	0.02*
Weight (Kg)	73.68±7.7	77.61±12.5	77.72±12.4	0.47
Height (cm)	167.89±9.2	164.33±8.3	166.09±11	0.51
BMI (kg/cm2)	26.25±3	28.71±4.1	28.54±3.6	0.09
Duration of diabetes (years)		4.8±2	15.5±7	
Medication (insulin+drugs/drugs) (N of cases)		1/17	3/9	
PAI (sedentary/poor/fair) (%)	63.2, 26.3, 10.5	66.7, 27.8, 5.6	45.5, 54.5,0	0.83
ABI	1.17±0.08	1.22±0.09	1.14±0.14	0.43
HbA1c (%)	4.7±0.8	7.02±1.5	7.3±1.4	0.000†
FBS (mmol/l)	91±12.6	141.65±36.9	160.91±31.4	0.000†
Blood sugar (mmol/l)	114.05±17.7	167.39±44.23	205.20±84.24	0.000†

Data are mean±SD.

* : Post Hoc between health and long-term T2DM groups,

† : Post Hoc between healthy and long-term T2DM and short-term T2DM groups.

FBS: fasting blood sugar

The CMPT were less in both diabetic groups in comparison to healthy control group and in females compared to males in both extension and flexion movement. There was no significant difference between two diabetic groups (Table 2).

**Table 2:** Mean (SD) and P value of two-way ANCOVA of CMPT and two-way ANOVA of slope and fatigue index between three groups and two genders

	Gender	Healthy control (N=19)	short-term T2DM (N=18)	Long-term T2DM (N=12)	p-value		

					Group	Gender	Gender×Group
**CMPT EXT (Newton meter)**	Male	1.43(.24)	.99(.3)	.8(.22)	0.000*	**0.000**	0.053
	Female	.81(.27)	.66(.2)	.53(.09)			
**CMPT FLEX (Newton meter)**	Male	.79(.22)	.56(.15)	.5(.11)	0.001*	**0.002**	0.61
	Female	.56(.13)	.43(.16)	.36(.09)			
**EXT fatigue index**	Male	60.53(16)	58.14(13)	51.17(12)	0.31	**0.014**	0.85
	Female	50.13(14)	45.08(12)	43.83(12)			
**FLEX fatigue index**	Male	63.91(9)	63.11(8)	73.11(9)	**0.008§**	**0.05**	0.34
	Female	63.01(7)	52.7(13)	67.18(4)			
**EXT SLOPE**	Male	-.88(.5)	-.89(.4)	-.94(.5)	0.94	0.12	0.88
	Female	-1.13(.6)	-1.23(.3)	-1.08(.4)			
**FLEX SLOPE**	Male	-.73(.4)	-.75(.4)	-.39(.2)	**0.006†**	0.39	0.49
	Female	-.74(.2)	-1.02(.4)	-.4(.1)			

Data are mean (SD) in the descriptive columns and p-value in the analytical columns,

* : healthy control and both diabetic groups,

§ : Post Hoc was significant between long-term and short-term T2DM and

† : Post Hoc was significant between long-term T2DM and two other groups.

M: men, W: women, CMPT: concentric maximum peak torque, EXT: extension, FLEX: flexion.

Table 2 also showed that the slope of relative peak torques of flexion movement was more downward in the short-term T2DM and healthy control groups than the long-term T2DM group. The flexion fatigue index in the short-term T2DM was lower than the long-term T2DM group, which means that the patients of long-term T2DM group were more resistant to fatigue than the two other groups in the flexion movement. The effect of gender showed that men had a higher extension fatigue index than women. The significant interaction was not found between grouping and gender. In general, the extension fatigue index was significantly lower than flexion fatigue index (F=4.02, 2-tailed significant=0.000).

The effect of grouping on MPF was showed that only the MPF of long head of biceps femoris muscle was different between groups which interacted with gender. It means the MPF of biceps femoris of women of the short-term T2DM group was much higher than women of healthy control subjects. The comparison of initial and final EMG demonstrated that MPF was significantly decreased during isokinetic protocol in all muscles, independently to groups and sex except medial hamstring. The medial hamstring showed an interaction effect between time and groups. The decrease of MPF of medial hamstring in the long-term T2DM was significantly lower than two other groups (Table 3).

**Table 3:** Mean (SD) and P value of three-way mixed model ANOVA of MPF between three groups and two sexes, initial and final part of isokinetic protocol

EMG		Time	Gender	Groups			P Value						

				Healthy control (N=19)	short-term T2DM (N=18)	Long-term T2DM (N=12)	Time	Group	Gender	Group × Gender	Time × Group	Time × Gender	Time × Group × Gender
**Mean power frequency**	**Vastus Lat.**	**Initial**	**Male**	66.52(7)	67.65(14)	71.95(12)	**0.000**	0.53	0.83	0.93	0.77	0.82	0.17
			**Female**	65.92(14)	72.35(9)	69.41(17)							
		**Final**	**Male**	54.25(7)	58.95(15)	57.4(9)							
			**Female**	55.39(10)	59.21(8)	58.7(14)							
	**Vastus Med.**	**Initial**	**Male**	76.28(9)	72.51(14)	74.74(3)	**0.000**	0.43	0.32	0.35	0.57	0.93	0.60
			**Female**	73.87(14)	82.31(8)	76.65(22)							
		**Final**	**Male**	54.91(3)	58.08(12)	58.38(10)							
			**Female**	56.57(7)	65.68(11)	57.68(10)							
	**Biceps femoris**	**Initial**	**Male**	85.73(12)	92.42(29)	97.48(26)	**0.000**	0.025*	0.15	**0.032**	0.24	0.66	0.98
			**Female**	86.38(13)	122.94(9)	87.49(23)							
		**Final**	**Male**	62.63(12)	64.02(10)	81.48(20)							
			**Female**	66.54(7)	95.84(11)	74.53(13)							
	**Med. hamstring**	**Initial**	**Male**	75.9(9)	92.99(21)	74.73(8)	**0.000**	0.20	0.30	0.49	**0.041**	0.11	0.72
			**Female**	83.86(18)	88.19(19)	74.63(14)							
		**Final**	**Male**	52.86(7)	63.06(20)	64.69(7)							
			**Female**	67.9(16)	70.31(14)	67.2(14)							

Data are mean (SD) in the descriptive columns and p-value in the analytical columns,

* : Post Hoc was significant between healthy control and short-term T2DM.

The effect of grouping on RMS was seen in all muscles in which the healthy control had higher RMS than two diabetic groups. Although, the interaction between group and time was significant in the vastus lateralis and vastus medialis. The RMS of medial hamstring was increased significantly in interaction with gender after fatigue protocol. Increase in the vastus lateralis RMS interacted with grouping and gender. It means that increase in the vastus lateralis RMS was higher in men than women, and in the healthy control and long-term T2DM group than short-term T2DM group during isokinetic protocol. The results also showed a decrease in the vastus medialis RMS of long-term T2DM group at the end of repetitions, while other groups showed an increase in RMS (Table 4).

**Table 4:** Mean (SD) and P value of three-way mixed model ANOVA of RMS between three groups and two sexes, initial and final part of isokinetic protocol

EMG		Time	Gender	Groups			P Value						

				Healthy control (N=19)	short-term T2DM (N=18)	Long-term T2DM (N=12)	Time	Group	Gender	Group × Gender	Time × Group	Time × Gender	Time × Group × Gender
**Root mean squre (µV)**	**Vastus Lat.**	**Initial**	**Male**	353.21(98)	264.66(110)	233.15(79)	**0.000**	**0.006‡**	**0.002**	0.59	**0.034**	**0.036**	0.72
			**Female**	240.48(118)	204.71(161)	97.5(33)							
		**Final**	**Male**	410.78(97)	284.24(110)	276.14(100)							
			**Female**	273.78(140)	213.42(180)	109.56(30)							
	**Vastus Med.**	**Initial**	**Male**	210(62)	158.98(45)	172.74(51)	0.083	**0.021§**	**0.000**	0.59	**0.045**	0.36	0.60
			**Female**	120.97(49)	104.19(50)	78.67(29)							
		**Final**	**Male**	244.98(80)	182.19(70)	158.78(42)							
			**Female**	135.02(63)	111.82(72)	71(22)							
	**Biceps femoris**	**Initial**	**Male**	177.43(75)	97.85(41)	123.12(44)	0.60	*0.017	0.15	0.72	0.72	0.76	0.83
			**Female**	137.21(29)	97.92(57)	74.79(31)							
		**Final**	**Male**	180.22(87)	95.42(35)	118.91(31)							
			**Female**	137.64(32)	82.17(51)	75.68(38)							
	**Med. hamstring**	**Initial**	**Male**	259.56(94)	153.17(78)	142.24(47)	0.089	**0.002‡**	**0.002**	0.75	0.46	**0.039**	0.15
			**Female**	163(64)	113.89(64)	75.75(6)							
		**Final**	**Male**	272.33(104)	198.42(115)	146.76(57)							
			**Female**	164(75)	106.18(64)	75.98(22)							

Data are mean (SD) in the descriptive columns and p-value in the analytical columns,

* : Post Hoc was significant between healthy control and both diabetic,

‡ : Post Hoc was significant between healthy control and long-term T2DM and

§ : Post Hoc was significant between long-term T2DM and two other groups.

The correlation analysis showed that both flexion and extension CMPT had significant negative correlation with all blood variables such as FBS, HBA1c, blood sugar during test session (R = -0.35 to -0.54. and p-value of 0.0001 to 0.013). No significant correlation was observed between blood variables, slope, FI and EMG. CMPT and initial RMS of all muscles were correlated positively (R = 0.48 to 0.75 and p-value of 0.0001-0.008).

## 4. Discussion

The current study tried to evaluate effect of short and long-term T2DM on muscle strength and endurance of knee muscles during concentric contraction of knee extension and flexion by isokinetic dynamometer and EMG recording. T2DM patients had weaker knee muscles. The study showed that CMPT of knee extension and flexion was significantly greater in healthy control subjects than both diabetes groups, while no significant difference was found between long and short-term T2DM subjects. Also, males showed greater CMPT in both motions than females and CMPT negatively correlated with blood sugar parameters. The analysis of fatigue indices showed the short-term T2DM patients were fatigued earlier than healthy control but long-term T2DM patients were more resistant to fatigue than health control. Women were subjected to more fatigue than men. During isokinetic protocol, the decrease of mean power frequency of all muscles was independent to grouping except the medial hamstring. The RMS of medial hamstring was significantly increased in a gender-dependent manner. It means that the changes in RMS were not significant in women. Moreover the RMS of vastus medialis showed decrease in long-term T2DM while increase in other groups.

In this study, patients were assigned to two groups: patients with less than 10 years and patients with equal or more than 10 years of T2DM from first diagnosis. This category selected based on finding of previous studies and existing facilities. Previous studies showed that patients who had T2DM for 6 years exhibited different mitochondrial activity in the progressive aerobic exercise in comparison with patients that had diabetes for more than 12 years. The short-term diabetes group (less than 6 years of diabetes) had longer phosphocreatine recovery half time (PCr half-time) than the BMI-matched healthy group, whereas IMCL (intramyocellular lipid concentration) content was similar. The long-term diabetes group (more than 12 years of diabetes) had a higher IMCL content with no significant difference in the mitochondrial function, i.e. PCr and ADP recovery times (De Feyter et al., 2008; Schrauwen-Hinderling et al., 2007). Therefore we categorized the patients to long and short-term T2DM near to previous studies.

Several studies proved that diabetes duration, but not diabetes duration and endurance indices, was negatively correlated with strength of lower limb (Andersen et al., 1996; Andreassen, Jakobsen, & Andersen, 2006; Kalyani et al., 2013; Park et al., 2007). The current study confirmed previous studies. In the study of Andersen et al. (1998) T2DM with more than 20 years of disease were resistant to fatigue in comparison to health subjects, which were in accordance with endurance response of long-term T2DM group in the current study. In the study of IJzerman et al., which the duration of diabetes in patients has not been reported (IJzerman, 2012), were matched to our findings of the short-term T2DM group. It seems that the opposite results of two above mentioned studies may be related to the duration of diabetes that was covered in this study. Different responses of knee flexor and extensor motion may be related to the test conditions and a different distribution of muscle fibers (Andersen, 1998; IJzerman, 2012).

Fatigue causes decreases of MPF (Babault, Desbrosses, Fabre, Michaut, & Pousson, 2006; Gonzalez-Izal, Malanda, Gorostiaga, & Izquierdo, 2012). Decreased MPF is a sign of less synchronization and muscle fiber conduction velocity may be due to the decrease of neurotrophic factors (Babault et al., 2006). We found that MPF diminished after fatigue but it was less significant in long-term T2DM than other groups in medial hamstring muscle. Some researches revealed results that did not confirmed our results. It may be the history of diabetes effect on EMG frequency. Andreassen et al found that deficit in neurotrophic factors in T2DM were higher than health subject (Andreassen, Jakobsen, Flyvbjerg, & Andersen, 2009). At the vastus lateralis, but not the biceps brachii, the muscle fiber conduction velocity was reduced in sedentary diabetic patients. The muscle strength was also declined in sedentary diabetic patient in comparison to trained diabetic patients and health subjects (Sacchetti et al., 2013). Another study showed that in the diabetic patients, the conduction velocity of tibialis anterior was more than vastus lateralis (Sacchetti et al., 2013). It seems that muscles of the lower limb respond differently to the effects of diabetic neuropathy.

Although RMS did not follow force decrease due to fatigue (Gerdle, Larsson, & Karlsson, 2000), but it correlate with force value which supports the relationship between muscle force and RMS (Disselhorst-Klug, Schmitz-Rode, & Rau, 2009; Luera, Stock, & Chappell, 2014).

Regarding to the test condition, knee extensors resist against both the load of the lever arm and gravity, while the knee flexors only opposed to the lever arm. Therefore it is expected that the extensors worked harder and were more susceptible to greater force reduction than flexors. This is the reason of lower fatigue index in the knee extension than flexion motion in all subjects. Although, the knee flexor and extensor muscles consist of approximately equal percentage of fast and slow muscle fibers, but the percent of slow fibers is dominant in the quadriceps (Lexell, Henriksson-LarsÉN, & SjÖStrÖM, n. d.) and fast fibers are dominant in hamstring muscles (Dahmane, Djordjevic, & Smerdu, 2006; Garrett, Califf, & Bassett, 1984). Therefore, the hamstring muscle is less resistant to fatigue and has more glycolytic capacity for anaerobic activity than the quadriceps (Zierath & Hawley, 2004). Diabetic muscles also used glycolytic stores and type 2 muscle fiber (MÅrin et al., 1994; Oberbach et al., 2006), which might be the reason of more resistance to anaerobic fatigue in the knee flexors of long-term T2DM.

As revealed, fast-twitch, type II fibers are dominant the in T2DM. Moreover, the oxidative and glycolytic enzymes of all types of muscles fibers and the density of GLUT4 in type 2 muscle fibers were increased in the T2DM (MÅrin et al., 1994; Nyholm et al., 1997; Oberbach et al., 2006). Therefore, long-term changes in muscle fiber distribution due to diabetes provide some insight to understand the reason of fatigue response in T2DM. However, it was established that the duration of diabetes were the most important risk factors for the development of neuromuscular complications (Cameron & Cotter, 1994; Park et al., 2007; Partanen et al., 1995) but it is not clear that how long-term changes of muscles and their enzymes modulate the energy to produce the required strength and endurance (Gaster, Poulsen, Handberg, Schrøder, & Beck-Nielsen, 2000; Schiaffino & Reggiani, 2011; Zierath & Hawley, 2004). In order to compensate the loss of type 1 muscle fibers and preserve its performance, the oxidative and glycolytic capacity of the type 2 muscle fibers will gradually rise (Oberbach et al., 2006). Indeed, according to some studies (Andersen, 1998; Andreassen, et al., 2006; Chattopadhyay et al., 2011; De Feyter et al., 2008; Halvatsiotis et al., 2002; Hatef, Bahrpeyma, & Tehrani, 2014; Schrauwen-Hinderling et al., 2007), it is suggested that compensatory mechanism related to long-term diabetes may occur in order to increase the muscle capacity and to preserve the performance in the muscles like knee flexors which consists predominantly of type 2 fibers during intensive short-term exercise.

This study has some limitations which have to be pointed out. The sample size for each patient group was not calculated. Although some of basic confounding factors such as gender, BMI, PAI and ABI were controlled in this study but the neuropathy rank-sum score was not measured. Long-term T2DM patients were also older than control subjects. To solve this problem, the analysis of covariance was carried out for the age factor.

## 5. Conclusion

In conclusion, gender, BMI, ABI and PAI-matched healthy control group exhibited greater knee extension and flexion CMPT and RMS of knee muscles than both long and short-term type 2 diabetes groups. The long-term T2DM group revealed less force decline in flexor motion and MPF of medial hamstring than short-term T2DM and even healthy control groups during intensive short-term isokinetic protocol. The investigation of muscle fatigue following aerobic and moderate endurance exercise in the diabetic population with varying duration of disease is a potential direction for future researches.
